# An Acid-Responsive Fluorescent Molecule for Erasable Anti-Counterfeiting

**DOI:** 10.3390/molecules29184335

**Published:** 2024-09-12

**Authors:** Jiabao Liu, Xiangyu Gao, Qingyu Niu, Mingyuan Jin, Yijin Wang, Thamraa Alshahrani, He-Lue Sun, Banglin Chen, Zhiqiang Li, Peng Li

**Affiliations:** 1 School of Chemical Engineering and Technology, Hebei University of Technology, Tianjin 300130, China; 202221501035@stu.hebut.edu.cn (J.L.); 202211501008@stu.hebut.edu.cn (Q.N.); 2 Shanghai Key Laboratory of Molecular Catalysis and Innovative Materials, Fudan University, Shanghai 200438, China; 22110220021@m.fudan.edu.cn (X.G.); 21300220007@m.fudan.edu.cn (Y.W.); 3 College of Chemistry and Materials Science, Hebei Normal University, Shijiazhuang 050024, China; jinmingyuan@stu.hebtu.edu.cn (M.J.); heluesun@hebtu.edu.cn (H.-L.S.); 4 Department of Physics, College of Science, Princess Nourah bint Abdulrahman University, Riyadh 11671, Saudi Arabia; thmalshahrani@pnu.edu.sa; 5 Fujian Provincial Key Laboratory of Polymer Materials, College of Chemistry & Materials Science, Fujian Normal University, Fuzhou 350007, China; banglin.chen@fjnu.edu.cn

**Keywords:** aggregation-induced emission enhancement (AIEE), stimuli-responsive luminescence, tetraphenylethylene (TPE), diaminotriazine (DAT), anti-counterfeiting

## Abstract

A tetraphenylethylene (TPE) derivative, TPEPhDAT, modified by diaminotriazine (DAT), was prepared by successive Suzuki–Miyaura coupling and ring-closing reactions. This compound exhibits aggregation-induced emission enhancement (AIEE) properties in the DMSO/MeOH system, with a fluorescence emission intensity in the aggregated state that is 5-fold higher than that of its counterpart in a dilute solution. Moreover, the DAT structure of the molecule is a good acceptor of protons; thus, the TPEPhDAT molecule exhibits acid-responsive fluorescence. TPEPhDAT was protonated by trifluoroacetic acid (TFA), leading to fluorescence quenching, which was reversibly restored by treatment with ammonia (on–off switch). Time-dependent density functional theory (TDDFT) computational studies have shown that protonation enhances the electron-withdrawing capacity of the triazine nucleus and reduces the bandgap. The protonated TPEPhDAT conformation became more distorted, and the fluorescence lifetime was attenuated, which may have produced a twisted intramolecular charge transfer (TICT) effect, leading to fluorescence redshift and quenching. MeOH can easily remove the protonated TPEPhDAT, and this acid-induced discoloration and erasable property can be applied in anti-counterfeiting.

## 1. Introduction

Stimuli-responsive luminescent materials are materials with intelligent-response characteristics that can change their fluorescent color by external stimuli such as force [1,2], temperature [3,4,5], pH [6,7], light [8,9,10], magnetic field [11], etc. [12]. Among them, acid–base stimulus-responsive fluorescent materials are crucial in anti-counterfeiting and security. Adding acid–base stimulus-responsive fluorescent materials to a label changes the color or pattern of the label when exposed to acid. This can protect products from counterfeiting and tampering [13,14]. A series of acid–base stimulus-responsive molecules have been reported [14,15,16], and such molecules’ excellent fluorescence-switching properties are expected to be developed in anti-counterfeiting.

The controlled erasure of fluorescent molecules is crucial for securely transmitting encrypted information. However, most current research on erasable materials still relies on the attenuation of fluorescent signals. The development of molecular materials that can be removed with common solvents presents a more convenient method for tamper-resistant removal. Nevertheless, this method of conveniently erasing information still requires further improvement [17,18].

Most systems discussed so far suffer from the aggregation-caused quenching (ACQ) effect, which is very limited in practical applications involving solid substrates. Unlike conventional fluorescent materials, which are subject to concentration quenching, some fluorescent molecules have extraordinary fluorescence emission properties, known as aggregation-induced emission enhancement (AIEE). As widely reported [19,20,21], AIEE is a phenomenon in which fluorescent molecules emit weakly in the monomeric state and are greatly enhanced in the aggregated state. Therefore, introducing AIEE properties into smart-response anti-counterfeiting materials is an ideal strategy to solve the limitations of ACQ [22,23].

Therefore, we have designed a tetraphenylethylene (TPE)-based molecule decorated with diaminotriazine (DAT) groups, which shows strong AIEE properties and can hardly dissolve in any common solvent. However, the molecule can easily react with various organic and inorganic acids by protonating the N atoms in the triazine rings. Over 90% or more fluorescence diminution occurs upon reaction, while TPEPhDAT, after protonation, can be quickly removed with common MeOH solvents. We demonstrate that this material can be processed into paper with encrypted information for anti-counterfeiting applications by solution methods. This novel design not only improves the security of anti-counterfeiting technology but also provides a new solution for the confidentiality and controllability of information.

## 2. Results and Discussion

### 2.1. Synthesis and Structural Elucidation of TPEPhDAT

**Scheme 1:**
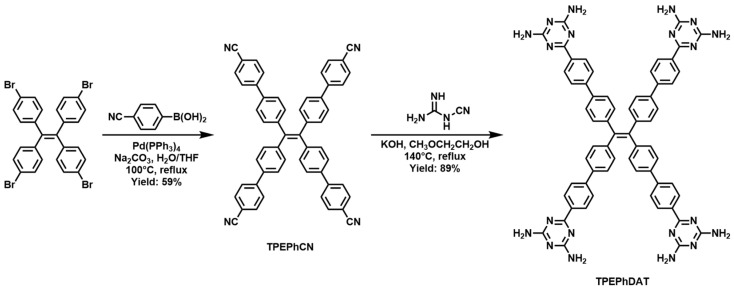
Synthetic route of TPEPhDAT.

The synthetic route of TPEPhDAT was obtained by modification of previous reports [24,25], the synthetic route of which is shown in Scheme 1. 1,1,2,2-tetrakis(4-bromophenyl)ethene and 4-cyanophenylboronic acid underwent Suzuki–Miyaura coupling to obtain extended TPEPhCN. The yellowish TPEPhDAT could be easily obtained in an 80% yield by reacting the corresponding nitrile with dicyandiamide. The structure of TPEPhDAT was characterized and confirmed by ^1^H NMR (Figures S1 and S2), ^13^C NMR (Figures S3 and S4), and liquid chromatography–mass spectrometry (LC-MS, Figure S5).

### 2.2. AIEE Characteristic of TPEPhDAT

**Figure 1:**
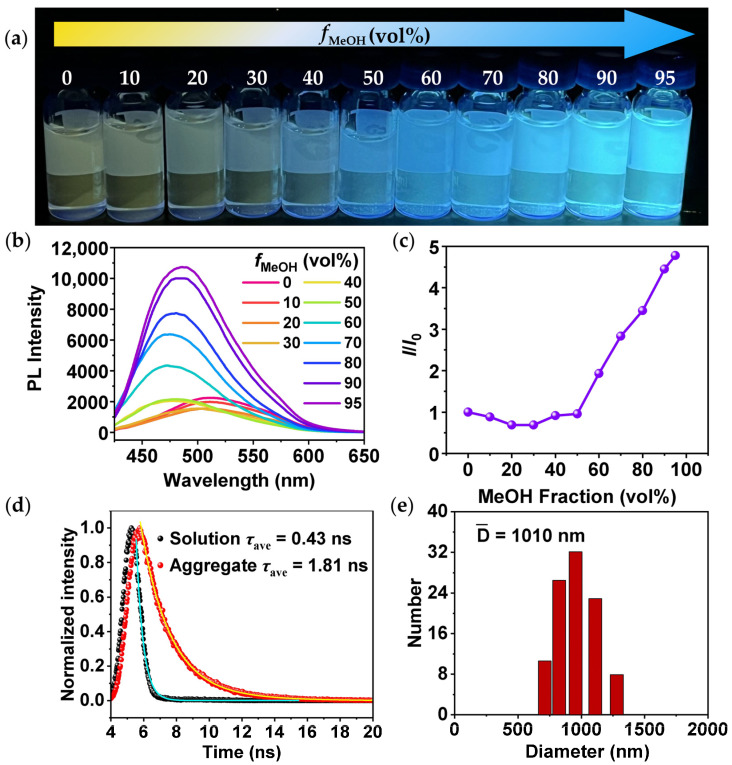
(**a**) In the DMSO/MeOH solvent mixture, the photograph of the volume fraction of MeOH (*f*_MeOH_) increasing from 0 vol% to 95 vol% (under UV light); (**b**) fluorescence spectra of TPEPhDAT in DMSO/MeOH mixtures with different *f*_MeOH_ (concentration: 10 μM; *λ*_ex_: 380 nm); (**c**) plot of relative PL intensity (*I*/*I*_0_) vs *f*_MeOH_; (**d**) time-resolved decay curves of solution (*f*_MeOH_ = 0 vol%) and aggregate (*f*_MeOH_ = 95 vol%) at *λ*_ex_ = 380 nm; (**e**) hydrodynamic radius distribution of aggregates when *f*_MeOH_ = 95 vol%.

The AIEE properties of TPEPhDAT were explored in the DMSO/MeOH system (Figure 1a). In this system, DMSO is a good solvent, and MeOH is a poor solvent. The luminescence of TPEPhDAT is extremely weak in pure DMSO. When the volume fraction of MeOH (*f*_MeOH_) was increased from 0 vol% to 30 vol%, the fluorescence emission spectra were continuously blue-shifted, and the intensity of the emission peaks decreased slightly. This particular fluorescence change has been identified in existing reports [26,27,28], and this phenomenon can be attributed to the suppression of the proximity effect (SOPE) [29,30]. When *f*_MeOH_ was greater than 30 vol%, the intensity of the emission peaks was gradually enhanced, reaching a maximum at *f*_MeOH_ of 95 vol% (Figure 1b). Compared with the pure DMSO system, the fluorescence intensity increased about 5-fold (Figure 1c). Additionally, the fluorescence spectra of the aggregates were blue-shifted compared with that of the solution (Figure 1b). The above phenomenon results from the joint action of the solvation effect and the AIEE effect [31,32].

In dilute DMSO solution, the benzene ring rotor of TPE undergoes dynamic intramolecular rotation, and the excited-state energy is dissipated in the form of nonradiative leaps, resulting in weak luminescence. As the *f*_MeOH_ increases, the molecule appears aggregated, and the rotation of the benzene ring rotor is spatially limited. The restricted intramolecular rotation (RIR) suppresses the abovementioned nonradiative decay [33,34], and the excited-state molecules can only return to the ground state by radiative decay, significantly enhancing the fluorescence. When *f*_MeOH_ = 95 vol%, the absorption in the visible spectral region is small (Figure S6), which is consistent with the behavior of the aggregated state [35]. Figure 1d and Table S1 show the fluorescence lifetime decay curves of TPEPhDAT in DMSO solution and the DMSO/MeOH mixture (*f*_MeOH_ = 95 vol%). The *τ*_ave_ value of TPEPhDAT increases from 0.43 ns to 1.81 ns as the *f*_MeOH_ in the mix is increased from 0 vol% to 95 vol%. The longer fluorescence lifetime suggests that TPEPhDAT is more immobilized and aggregated [36,37]. In the meantime, when *f*_MeOH_ = 95 vol%, aggregates with a hydrodynamic diameter of about 1 μm appeared, as confirmed by dynamic light scattering (DLS, Figure 1e) and scanning electron microscopy (SEM, Figure S7).

### 2.3. Acidochromism

**Figure 2:**
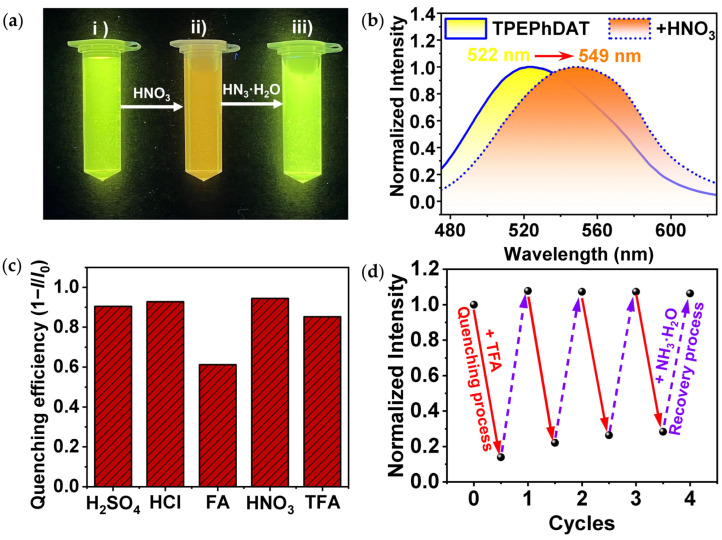
(**a**) Pictures of acid-responsive fluorescence color changes (2 mg/mL aqueous solution, under UV light) of TPEPhDAT (i) in its original state, (ii) after dropwise addition of HNO_3_, and (iii) after dropwise addition of ammonium hydroxide; (**b**) fluorescence spectra of TPEPhDAT under 350 nm excitation before and after dropwise addition of HNO_3_; (**c**) fluorescence quenching efficiency of TPEPhDAT with the addition of different acids; (**d**) fluorescence recovery cycle of TPEPhDAT to TFA: the red solid line indicates the quenching process and the purple dashed line indicates the recovery process.

The nitrogen atoms in the DAT moiety are considered effective proton acceptors [7,38,39]. Given this, we have investigated the possible acid-induced fluorescence quenching properties of TPEPhDAT in aqueous solution. In an aqueous dispersion of TPEPhDAT, an excess of HNO_3_ was added dropwise, and an immediate change in fluorescence was observed (Figure 2a). As shown in Figure 2b, the aqueous dispersion of TPEPhDAT showed bright yellow fluorescence with the maximum emission peak at 522 nm (*λ*_ex_ = 350 nm). In contrast, the dropwise addition of HNO_3_ completely eliminated the initial emission band at 522 nm, and a new peak at 549 nm corresponded to the orange emission. The fluorescence of TPEPhDAT+HNO_3_ was extremely weak, and the quenching rate reached 95%. In addition, we also discussed the acid-induced fluorescence discoloration properties of four acids, including HCl, trifluoroacetic acid (TFA), H_2_SO_4_, and formic acid (FA), in addition to HNO_3_ (Figure S8). The results showed that similar results were presented with HNO_3_, and the quenching efficiency of HCl, H_2_SO_4_, and TFA could reach more than 85%, while FA being less acidic, could reach a quenching efficiency of only 61% (Figure 2c). The results of the absolute quantum yield (*Φ*) tests closely agree with those of fluorescence quenching (Figure S9). In an aqueous solution, the *Φ* of TPEPhDAT can reach 57%, which decreases to different degrees after adding different acids, especially after adding HNO_3_. The *Φ* can be reduced to 6.9%. It has been proven that the fluorescence change of TPEPhDAT is caused by hydrogen ions (H^+^) rather than by anions (NO_3_^−^), and the stronger the acidity, the more pronounced the effect of acid discoloration becomes.

We attempted to treat acidified TPEPhDAT with ammonia. We found that adding excess ammonia fully restored the fluorescence, which was still fully restored after four cycles of repeated treatment with TFA and ammonia (Figure 2d). When the solution was treated with TFA and ammonia alternately, the fluorescence emission could be switched several times between “off and on” states. This implies that the reaction is entirely reversible.

### 2.4. Mechanism of TPEPhDAT Binding to Acid

**Figure 3:**
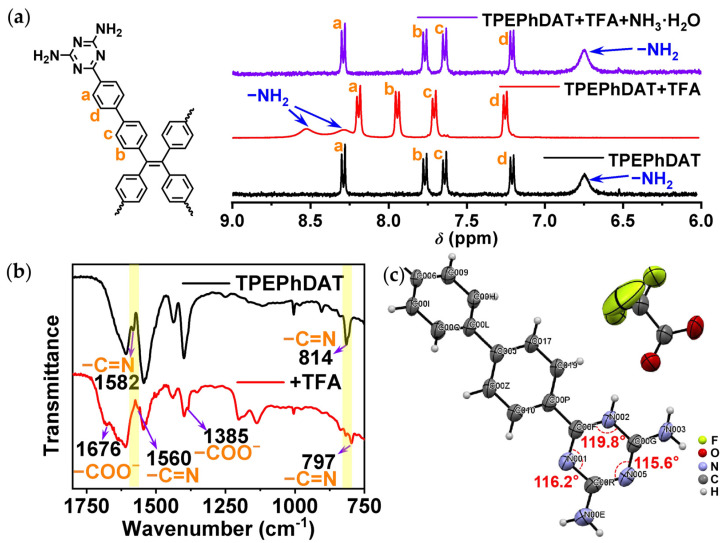
(**a**) Partial ^1^H NMR spectra of TPEPhDAT in DMSO-*d*_6_ after adding TFA and NH_3_·H_2_O; (**b**) FT-IR spectra of TPEPhDAT before and after protonation; (**c**) C−N−C internal angles for the protonated and unprotonated positions of the triazine ring (ellipsoids drawn at 50% probability).

To further understand the binding behavior at the molecular level, we performed ^1^H NMR measurements using TFA as an example. The two protons of −NH_2_ in TPEPhDAT show a single broad peak. In contrast, after protonation, the two protons shifted to a lower magnetic field compared to their positions before protonation and became two broad peaks (Figure 3a) [38]. This indicates that after protonation, the two −NH_2_ in the DAT group were no longer equivalent. To further illustrate the location of protonation, the calculated Gibbs free energy difference (∆*G*) and the corresponding Boltzmann population (Table S2) predict that protonation should occur at the location shown in Figure S10 [40].

This suggests that the protonation of TPEPhDAT occurs on the triazine ring rather than on −NH_2_. Fourier transform infrared (FT-IR) spectroscopy further confirms this. As shown in Figure 3b, the peaks at 1582 and 814 cm^−1^ significantly shift to 1560 and 797 cm^−1^ upon the addition of TFA. These changes are attributed to the deformation of the triazine ring from an ideal hexagonal shape upon protonation [41,42]. The peaks of deprotonated −COOH were also observed (1676 and 1385 cm^−1^) [43]. This indicates that upon the reaction of TFA with TPEPhDAT, the −COOH of TFA loses its proton and forms −COO^−^. The changes in FT-IR spectra provided evidence for the protonation of the triazine ring. The nitrogens on the triazine ring are more basic than those in the amine groups [42] because their lone pair electrons do not participate in conjugation, making them more vulnerable to proton attack. In addition, the strong electron absorption of the triazine ring causes the electronegativity of amino nitrogen to easily transfer to the triazine ring (Figure S11) [44], preventing the amino nitrogen from binding with protons.

Given this, we prepared co-crystals of TPEPhDAT with TFA by solvothermal methods (Figure S12). As can be seen from the crystal structure (Figure S13; Table S3), the protonation of TPEPhDAT occurs on the triazine ring rather than on −NH_2_. The protonated triazine ring deviates from the ideal ortho-hexagonal structure, and the C−N−C internal angle at the protonated position (C00F−N002−C00G: 119.8°) is much bigger than that at the unprotonated position (C00F−N001−C00R: 116.2°; C00G−N005−C00R: 115.6°), which is consistent with the literature reports (Figure 3c) [42]. This crystal structure provides a possible site for the binding of TPEPhDAT to acids.

### 2.5. Possible Mechanism of Acid-Induced Fluorescence Quenching in TPEPhDAT

**Figure 4:**
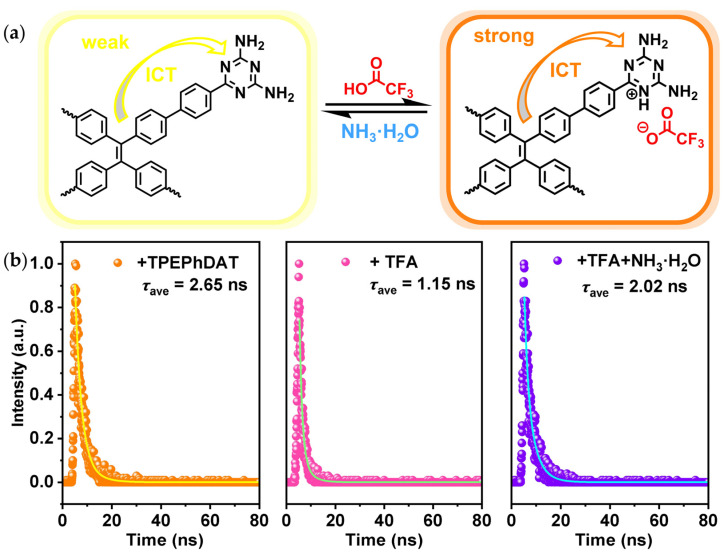
(**a**) Supposed sensing mechanism of TPEPhDAT to TFA; (**b**) time-resolved decay curves of TPEPhDAT after successive treatment by TFA and ammonia at *λ*_ex_ = 350 nm.

As can be seen in Figure 4a, the TPEPhDAT molecule has weak intramolecular charge transfer (ICT) properties. When TPEPhDAT reacts with TFA, the triazine ring exhibits a strong nucleophilic ability and can effectively attack the −COOH of TFA to generate the corresponding salt. Upon completion of protonation, the ICT effect of TPEPhDAT was enhanced, achieving a significant change in fluorescence from the initial yellow to orange [45,46]. Therefore, simple deprotonation methods can help restore the probe’s fluorescence properties. For example, TPEPhDAT can be deprotonated by adding ammonia, thus restoring the initial properties of the fluorescent probe.

To elucidate the chemical sensing system’s quenching mechanism, time-resolved fluorescence spectroscopy was utilized to investigate the quenching dynamics (Figure 4b and Table S4). The results revealed that the fluorescence lifetimes of TPEPhDAT were 2.65 ns and 1.15 ns before and after adding TFA, respectively, indicating a dynamic quenching process [47]. Furthermore, simple deprotonation treatment can restore the fluorescence of TPEPhDAT; for instance, dropping ammonia can restore the fluorescence lifetime to its initial state (2.02 ns).

**Figure 5:**
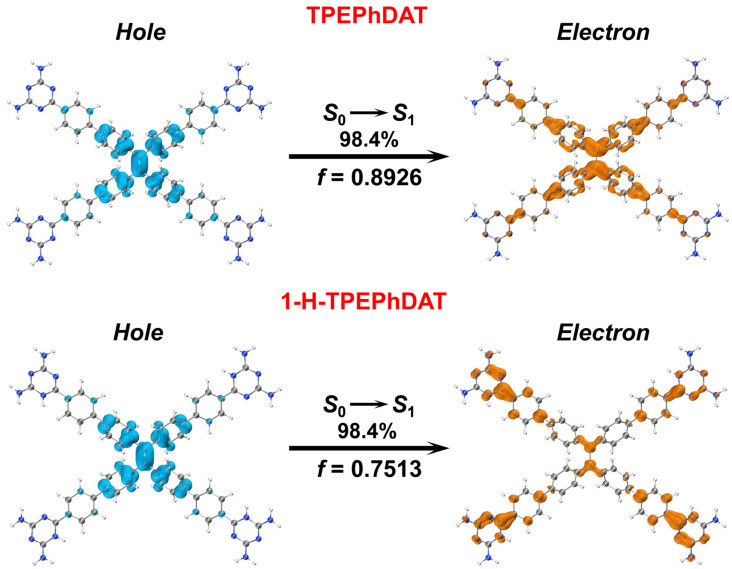
TDDFT calculations for TPEPhDAT and *S*_0_ → *S*_1_ electron–hole calculations. Protonation is adopted to simulate TFA stimulation, as previously reported.

In addition, we investigated the quenching mechanism of TFA on TPEPhDAT fluorescence by time-dependent density flooding theory (TDDFT) calculations. As shown in Figure 5, most of the “holes” of TPEPhDAT are localized on the TPE units of the backbone. Meanwhile, the “electrons” of TPEPhDAT are distributed throughout the molecular backbone. This result fits well with the weak ICT state of the *S*_0_ → *S*_1_ transition in the TPEPhDAT molecule. Upon protonation, it can be seen that the “holes” are mainly located on the TPE unit of 1-H-TPEPhDAT, which is similar to the TPEPhDAT probe. In contrast, during the *S*_0_ → *S*_1_ transition, the “electrons” are transferred to the triazine ring. As a result, a larger orbital spacing between the hole and the electron is observed, and the oscillator strength (*f*) correspondingly decreases from 0.8926 for TPEPhDAT to 0.7513 for 1-H-TPEPhDAT. The molecular energy is also simultaneously reduced upon protonation (Figure S14). These results indicate that 1-H-TPEPhDAT has stronger ICT properties [14,48]. This shows that the theoretical calculations agree with the sensing mechanism.

Generally, the molecule’s fluorescence lifetime increases with ICT enhancement [49]. However, the fluorescence lifetime decreases after protonation, and ICT enhancement increases the molecule’s dipole moment, which leads to a decrease in the energy difference between the excited and ground states. As a result, the excited-state electrons are more likely to return to the ground state via nonradiative pathways, thereby shortening the fluorescence lifetime. As shown in Figure S15, after the TPEPhDAT proton, the dihedral angle between the two phenyl planes increased from 34.25° to 40.88° (Figure S15). The molecule became more distorted, and a similar twisted intramolecular charge transfer (TICT) effect may have occurred [50], which increased the fluorescence nonradiative leap pathway and led to a shorter fluorescence lifetime.

### 2.6. Erasable Anti-Counterfeiting Applications

TPEPhDAT, as an AIEE-type fluorescent molecule, skillfully avoids the limitations of ACQ molecules on solid substrates. Therefore, we designed an anti-counterfeiting paper using solution processing. In addition, we took advantage of the reversible conversion of fluorescence of TPEPhDAT in acidic and alkaline environments and the excellent solubility of acidified TPEPhDAT in MeOH for the processing and erasure of encrypted information.

To confirm the utility of acid vapor fumigation for fluorescence discoloration under solid substrates, we obtained protonated TPEPhDAT samples by direct fumigation with acid vapor in a closed environment (Figure S16a) [51]. The phenomenon was similar to the change in water, with a nearly 95.7% fluorescence quench and a 38 nm redshift (Figure S16b). Ultraviolet–visible (UV–vis) spectra confirmed the redshift in absorption (Figure S16c). Meanwhile, to verify the durability of this fluorescent solid material, we tested its photo-stability and cycle stability. It was found that the fluorescence intensity of the material only decreased by 4% after continuous irradiation under ultraviolet light for 20 min (Figure S17a). The material can be fumigated with acid and alkali vapors for five cycles (Figure S17b), which confirms its good cyclic stability.

**Figure 6:**
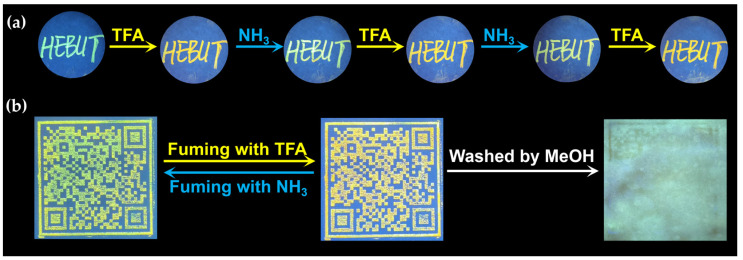
(**a**) The “**HEBUT**” pattern is treated alternately with acid and alkali vapors (under UV light); (**b**) pictures of reversible fluorescence switching of TPEPhDAT in acidic and alkaline environments and its erasure by MeOH (under UV light).

As illustrated in Figure 6a, the “HEBUT” pattern created with anti-counterfeit ink exhibits a simultaneous transition between greenish-yellow fluorescence and orange fluorescence when subjected to alternating acid and alkali stimuli. Following fumigation with TFA, the fluorescence of the 2D code printed using the same anti-counterfeiting ink was quenched, resulting in a color shift from yellow-green to orange. Notably, treatment with NH_3_ fully restored the fluorescence, thereby achieving reversible processing of encrypted information. Ultimately, the acidified TPEPhDAT can be effectively removed by rinsing with MeOH, facilitating the erasure of encrypted information under UV light (Figure 6b).

## 3. Materials and Methods

### 3.1. Materials

1,1,2,2-tetrakis(4-bromophenyl)ethene, tetrakis(triphenylphosphine)palladium [Pd(PPh_3_)_4_], 4-cyanophenylboronic acid, and 2-methoxyethanol were supplied by Adamas (Shanghai, China). Sodium carbonate (Na_2_CO_3_), potassium carbonate (K_2_CO_3_), sodium sulfate (Na_2_SO_4_), potassium hydroxide (KOH), dicyandiamide, sulfuric acid (H_2_SO_4_), dichloromethane (DCM), hydrochloric acid (HCl), methanol (MeOH), ethanol (EtOH), and silica gel were supplied by Greagent (Shanghai, China). Trifluoroacetic acid (TFA), ammonium hydroxide (NH_3_·H_2_O), nitric acid (HNO_3_), formic acid (FA), and tetrahydrofuran (THF) were supplied by Shanghai Hushi Chemical Co., Ltd. (Shanghai, China). Petroleum ether (PE) was supplied by Shanghai Titan Technology Co., Ltd. (Shanghai, China).

#### 3.1.1. Synthesis of Tetrakis [4-(4′-Cyanophenyl)phenyl]ethene (TPEPhCN)

In a 500 mL round-bottomed flask, 1,1,2,2-tetrakis(4-bromophenyl)ethene (2.5 g, 3.86 mmol), Na_2_CO_3_ (1 g, 9.43 mmol), 4-cyanophenylboronic acid (3.13 g, 21.28 mmol), and Pd(PPh_3_)_4_ (127 mg, 0.11 mmol) were added to a mixed solvent system consisting of THF (100 mL) and distilled water (50 mL). The mixture was stirred at 100 °C for 24 h under a nitrogen atmosphere. The mixture was cooled to room temperature. The mixture was quenched with 10% K_2_CO_3_ solution (30 mL). The mixture was extracted with DCM (50 mL × 3). The organic layer was collected, washed twice with saturated saline, and dried with anhydrous Na_2_SO_4_. The filtrate was filtered and concentrated under reduced pressure. The crude material was purified by silica gel column chromatography using PE and DCM as elution solvents (PE/DCM = 4:1). Removal of the solvent yielded a pale yellow solid (1.68 g, 2.28 mmol) with a 59% yield. ^1^H NMR (400 MHz, CDCl_3_) *δ* 7.67 (dd, *J* = 8.7 Hz, 16H), 7.42 (d, *J* = 8.4 Hz, 8H), 7.21 (d, *J* = 8.4 Hz, 8H).

#### 3.1.2. Synthesis of 6,6′,6″,6‴-(Ethene-1,1,2,2-tetrayltetrakis([1,1′-biphenyl]-4′,4-diyl))tetrakis(1,3,5-triazine-2,4-diamine) (TPEPhDAT)

In a 500 mL round-bottomed flask, tetrakis [4-(4’-cyanophenyl)phenyl]ethene (1 g, 1.36 mmol), KOH (90%, 0.75 g), and dicyandiamide (2.29 g, 27.2 mmol) were added to 2-methoxyethanol (40 mL) and stirred at 140 °C for 48 h under a nitrogen atmosphere. The mixture was then cooled to room temperature and poured into MeOH. The precipitated solid was filtered, washed with DCM and boiling water, respectively, and dried under vacuum at 90 °C to give a yellowish solid (1.3 g, 1.21 mmol) with a 89% yield. ^1^H NMR (400 MHz, DMSO-*d*_6_) *δ* 8.28 (d, *J* = 7.7 Hz, 8H), 7.75 (d, *J* = 7.9 Hz, 8H), 7.63 (d, *J* = 7.7 Hz, 8H), 7.20 (d, *J* = 7.6 Hz, 8H), 6.74 (s, 16H). ^13^C NMR (101 MHz, DMSO-*d*_6_/TFA, v:v = 9:1) *δ* 127.1, 127.4, 129.5, 130.1, 132.3, 137.3, 141.3, 144.0, 144.9, 161.0, 164.3. LC-MS *m*/*z* calculated for (C_62_H_48_N_20_) [M+H]^+^: 1073.4444; found: 1073.4509 (6 ppm).

#### 3.1.3. Synthesis of TPEPhDAT-TFA Single Crystalline

An amount of 6 mg of TPEPhDAT was added to a 2 mL vial containing 200 µL of TFA, followed by 80 µL of EtOH. The vial was capped and placed in an oven at 70 °C. After 12 h, the orange single crystals were collected for single-crystal X-ray diffraction analysis.

Details of the crystal data, data collection, structure solution, and refinement are shown in Table S3. CCDC 2345516 contains the supplementary crystallographic data for this paper. These data can be obtained free of charge via https://www.ccdc.cam.ac.uk/structures/? (accessed on 11 September 2024).

#### 3.1.4. Testing of Fluorescence Spectra of Acid-Induced Fluorescence Quenching of TPEPhDAT in Aqueous Solution

A homogeneous dispersion of 2 mg/mL was prepared by dispersing 40 mg of TPEPhDAT solid in 20 mL of water and sonicated for 20 min. The initial fluorescence emission spectra were recorded at an excitation wavelength of 350 nm. Take 2 mL of the above-configured dispersion with the cuvette, add 200 μL of 0.5 M HCl, HNO_3_, TFA, FA, or 0.25 M H_2_SO_4_ dropwise, shake uniformly, and then record the fluorescence emission spectra again at the excitation wavelength of 350 nm.

#### 3.1.5. The Fabrication of Anti-Counterfeiting Inks

An amount of 20 mg of TPEPhDAT was taken and ground thoroughly for 10 min. Then, 20 mL of EtOH was added and sonicated for 15 min to ensure the solids were well dispersed.

#### 3.1.6. Density Functional Theory (DFT) Calculations

All the DFT calculations were carried out using the Gaussian 16 (version C.01) package (Gaussian, Inc., Wallingford, CT, 2019, USA). The ground-state geometries were optimized under the B3LYP/6-31G(d,p) level. The DFT-D3 dispersion correction with BJ-damping was applied to correct the weak interaction and improve the calculation accuracy. The IEFPCM implicit solvation model was used to account for the solvation effect. The absorption properties were obtained by time-dependent density functional theory (TDDFT) with the B3LYP function at the same basis set level. Orbital energy level analysis and Electron excitation analysis were performed using Multiwfn software (version 3.8, dev) [52].

#### 3.1.7. Introducing and Discussing the Double-Exponential Fitting Function 

The double-exponential fitting function can be written as follows:

$$I=A_{1}\exp\left(-\frac{t}{\tau_{1}}\right)+A_{2}\exp\left(-\frac{t}{\tau_{2}}\right)$$

where *A*_1_ and *A*_2_ are constants, *t* is time, and *τ*_1_ and *τ*_2_ represent the decay lifetimes [53,54]. The average lifetime (*τ*_ave_) can be calculated as follows:

$$\tau_{\mathrm{ave}}=\frac{A_{1}\tau_{1}^{2}+A_{2}\tau_{2}^{2}}{A_{1}\tau_{1}+A_{2}\tau_{2}}$$

### 3.2. Methods

Solution ^1^H and ^13^C NMR spectra were collected by Bruker AVANCE III 400 MHz spectrometers (Bruker Corporation, Karlsruhe, Germany). The ^13^C NMR of TPEPhDAT was tested in DMSO-*d*_6_/TFA (9/1, v:v). The LC-MS spectrum was obtained using a Compact Bruker Scientific Instrument (Bruker Corporation, Karlsruhe, Germany). X-ray single crystal diffraction was collected by BL17B1 High Throughput Protein Crystallography Beamline in the Shanghai Synchrotron Radiation Facility and a Bruker D8 Venture MetalJet X-ray diffractometer equipped with a Photon II detector (Bruker Corporation, Karlsruhe, Germany). FT-IR spectra were collected by Thermo Fisher Nicolet iS10 (Thermo Fisher Scientific, Waltham, MA, USA). Photoluminescence spectra and lifetime were obtained on FLS1000 (Edinburgh Instruments, Livingstone, UK). Absolute PLQY was measured by a xenon lamp with an integrative sphere on FLS1000 (Edinburgh Instruments, Livingstone, UK). Solid UV–vis spectra were tested with a Lambda 650S UV–vis spectrophotometer (PERKIN ELMER, Waltham, MA, USA). Liquid UV–vis spectra were tested by a UV-9000S UV–vis spectrophotometer (METASH, Shanghai, China). DLS was examined on a Nano-ZS90 (Malvern Instruments, Malvern, England). SEM images were obtained on a LaB_6_ VEGA 3 XMU scanning electron microscope (TESCAN, Brno, Czech Republic) at an acceleration voltage of 200 V−30 kV.

## 4. Conclusions

This paper presents a DAT group-modified TPE molecule, TPEPhDAT, which was fabricated through Suzuki–Miyaura coupling and cyclization reactions. The DMSO/MeOH system demonstrated remarkable AIEE properties, and the luminescence intensity of its solid-state aggregates was approximately five times greater than that of the dilute solutions. As the triazine ring is prone to protonation, TPEPhDAT displays an obvious acid-induced fluorescence quenching behavior. The protonation occurs on the triazine ring rather than the −NH_2_ group, as verified by various means, such as ^1^H NMR, FT-IR, single crystals, etc. Reversible switching between yellow and orange-red fluorescence was accomplished by applying an alternate treatment of TFA with ammonia. The TDDFT calculations indicated that the electron-withdrawing capacity of the triazine unit is strengthened after TFA protonation, resulting in a reduction in the band gap and a change in the distribution of the electron cloud. Meanwhile, the protonated TPEPhDAT conformation became more distorted. It showed fluorescence lifetime decay, which predicts that the protonation process may be an obvious TICT process, directly leading to the redshift and quenching of fluorescence. Based on their acid-induced quenching behavior and the ability of MeOH to promptly remove them after protonation, they were processed into paper with encrypted information for anti-counterfeiting by the solution method. It can be observed that this property of reversible fluorescence switching under alternating acid–base stimulation renders the molecule potentially applicable in the field of information processing and anti-counterfeiting.

## Data Availability

The data presented in this study are available on request from the corresponding author.

## Supplementary Materials

The following supporting information can be downloaded at https://www.mdpi.com/article/10.3390/molecules29184335/s1, Figure S1. ^1^H NMR spectra of TPEPhCN (400 MHz, CDCl_3_); Figure S2. ^1^H NMR spectra of TPEPhDAT (400 MHz, DMSO-*d*_6_); Figure S3. ^13^C NMR spectra of TPEPhDAT (101 MHz, DMSO-*d*_6_/TFA, v:v = 9:1); Figure S4. Localized enlarged ^13^C NMR spectra of TPEPhDAT (101 MHz, DMSO-*d*6/TFA, v:v = 9:1); Figure S5. LC-MS spectra of TPEPhDAT; Figure S6. UV–vis absorption spectra of TPEPhDAT in solution (*f*_MeOH_ = 0 vol%) and aggregate (*f*_MeOH_ = 95 vol%); Figure S7. SEM pictures of aggregates at *f*_MeOH_ = 95 vol%; Figure S8. Emission spectra of TPEPhDAT dispersed in water before and after the addition of an aqueous solution of (a) H_2_SO_4_, (b) HCl, (c) FA, (d) TFA, and (e) HNO_3_; Figure S9. The absolute quantum yield of TPEPhDAT before and after the addition of various acids; Figure S10. There are two positions where protonation may occur: 1-H-TPEPhDAT and 3-H-TPEPhDAT; Figure S11. Electrostatic potentials mapped on isodensity surfaces of TPEPhDAT; Figure S12. Microscopic optical image for TPEPhDAT-TFA; Figure S13. Crystal molecular diagram of TPEPhDAT-TFA (ellipsoids drawn at 50% probability); Figure S14. Calculated absorption energy of bared and protonated TPEPhDAT monomer; Figure S15. Change in the dihedral angle between two phenyl planes before and after TPEPhDAT protonation; Figure S16. (a) Schematic diagram of TPEPhDAT fumigated with TFA. Fluorescence spectra (b) and UV–vis absorption spectra (c) of TPEPhDAT before and after fumigation with TFA; Figure S17. (a) The fluorescence emission intensity of TPEPhDAT after continuous UV irradiation for 20 min (*λ*_em_ = 521 nm); (b) cycle cycles of TPEPhDAT were treated with alternating TFA and NH_3_ vapors: the red solid line indicates the quenching process, and the purple dashed line indicates the recovery process. Table S1: The double-exponential fitting parameters of time-resolved PL decay curves for solution and aggregate states; Table S2: Calculated free energy differences and corresponding Boltzmann populations for different protonation sites (1-H-TPEPhDAT and 3-H-TPEPhDAT); Table S3: Crystallographic data of TPEPhDAT-TFA; Table S4: The double-exponential fitting parameters of time-resolved PL decay curves for TPEPhDAT, +TFA, and +TFA+NH_3_·H_2_O.
