# Supplementary material for: An Acid-Responsive Fluorescent Molecule for Erasable Anti-Counterfeiting
Source: Molecules. 2024 Sep 12;29(18):4335. doi: 10.3390/molecules29184335 (PMC11433798; doi:10.3390/molecules29184335)
Supplement: Supplementary file 1 [file molecules-29-04335-s001.zip › molecules-3153923-supplementary.pdf]

Supporting information

An Acid Responsive Fluorescent Molecule for Erasable Anti-Counterfeiting

Jiabao Liu,[1] Xiangyu Gao,[2] Qingyu Niu,[1] Mingyuan Jin,[3] Yijin Wang,[2] Thamraa Alshahrani,[4] He-Lue Sun,[3] Banglin Chen,[5] Zhiqiang Li,*[1] and Peng Li*[2]

1 School of Chemical Engineering and Technology, Hebei University of Technology, GuangRong Dao 8, Hongqiao District, Tianjin 300130 (China)

2 Shanghai Key Laboratory of Molecular Catalysis and Innovative Materials, Department of Chemistry, Fudan University, 2005 Songhu Road, Shanghai 200438 (China)

3 College of Chemistry and Materials Science, Hebei Normal University, Shijiazhuang 050024 (China)

4 Department of Physics, College of Science, Princess Nourah bint Abdulrahman University, Riyadh 11671 (Saudi Arabia)

5 Fujian Provincial Key Laboratory of Polymer Materials, College of Chemistry & Materials Science, Fujian Normal University, Fuzhou 350000 (China)

E-mails addresses: zhiqiangli@hebut.edu.cn (Zhiqiang Li), penglichem@fudan.edu.cn (Peng Li)

Table S1. The double exponential fitting parameters of time-resolved PL decay curves for solution and aggregate.

	A_1(%)	τ_1(ns)	A_2(%)	τ_2(ns)	τ_{ave}(ns)
Solution	49.76	0.43	50.24	0.43	0.43
Aggregate	97.35	1.74	2.65	4.50	1.81

Table S2. Calculated free energy differences and corresponding Boltzmann populations for different protonation sites (1-H-TPEPhDAT and 3-H-TPEPhDAT).

	ΔG (kcal/mol)	%pop
1-H-TPEPhDAT	0	100
3-H-TPEPhDAT	7.56	0

Table S3. Crystallographic data of TPEPhDAT-TFA.

Identification code	TPEPHDAT2_0m_a_pl
CCDC Number	2345516
Empirical formula	$C_{70}H_{54}F_{12}N_{20}O_8$
Formula weight	1531.33
Temperature/K	273.15
Crystal system	monoclinic
Space group	$C2/c$
a/Å	26.610(13)
b/Å	15.938(7)
c/Å	21.042(8)
α/°	90
β/°	108.691(13)
γ/°	90
Volume/Å^3	8453(6)
Z	4
ρ_{calc} g/cm^3	1.203
μ/mm^{-1}	0.1
F(000)	3144
Crystal size/mm^3	0.1 × 0.1 × 0.1
Radiation	MoKα (λ = 0.71073)
2θ range for data collection/°	3.024 to 50.056
Index ranges	$-31 \le h \le 31$, $-18 \le k \le 18$, $-25 \le l \le 25$
Reflections collected	49740
Independent reflections	7123 [R_{int} = 0.0531, R_{sigma} = 0.0406]
Data/restraints/parameters	7123/0/497
Goodness-of-fit on F^2	1.089
Final R indexes [$I \geqq 2\sigma(I)$]	R_1 = 0.0900, wR_2 = 0.2843
Final R indexes [all data]	R_1 = 0.1016, wR_2 = 0.3009
Largest diff. peak/hole / e Å^{-3}	0.57/-0.41

Table S4. The double exponential fitting parameters of time-resolved PL decay curves for TPEPhDAT, +TFA and $+TFA+NH_3{\cdot}H_2O$.

	A_1(%)	τ_1(ns)	A_2(%)	τ_2(ns)	τ_{ave}(ns)
TPEPhDAT	69.10	1.99	30.90	4.14	2.65
+TFA	97.01	1.08	2.99	3.35	1.15
$+TFA+NH_3{\cdot}H_2O$	75.33	1.39	24.67	3.94	2.02

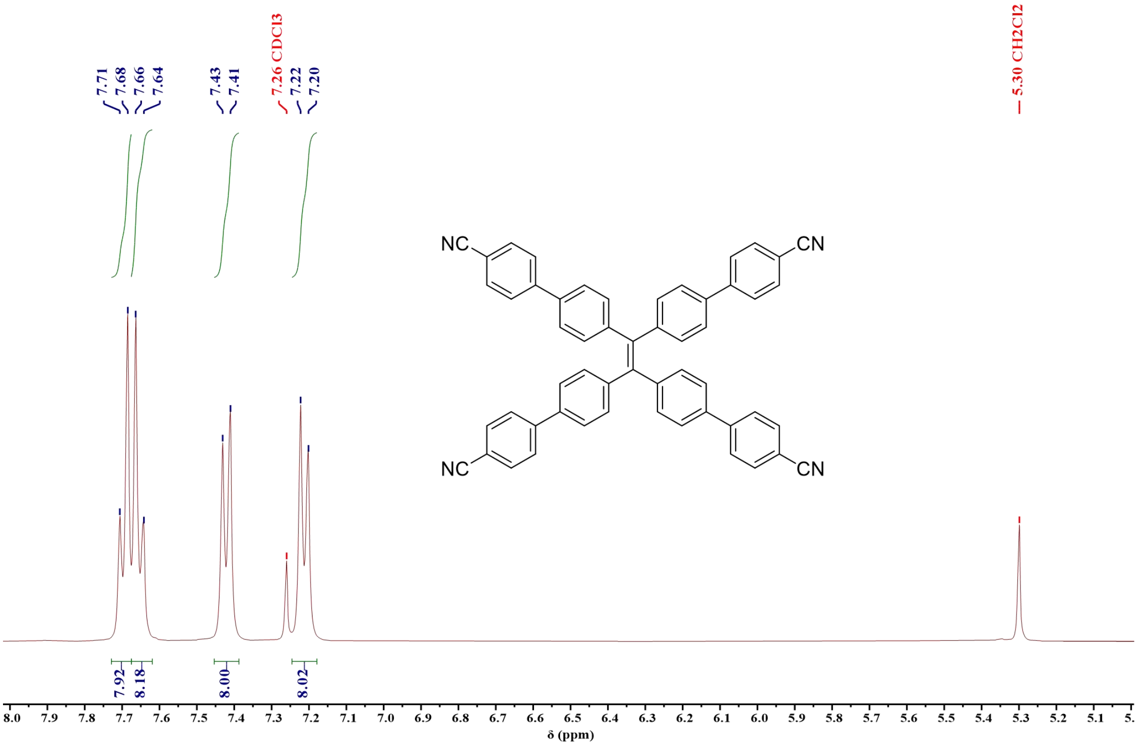

Figure S1. ^{1}H NMR spectra of TPEPhCN (400 MHz, $CDCl_3$).

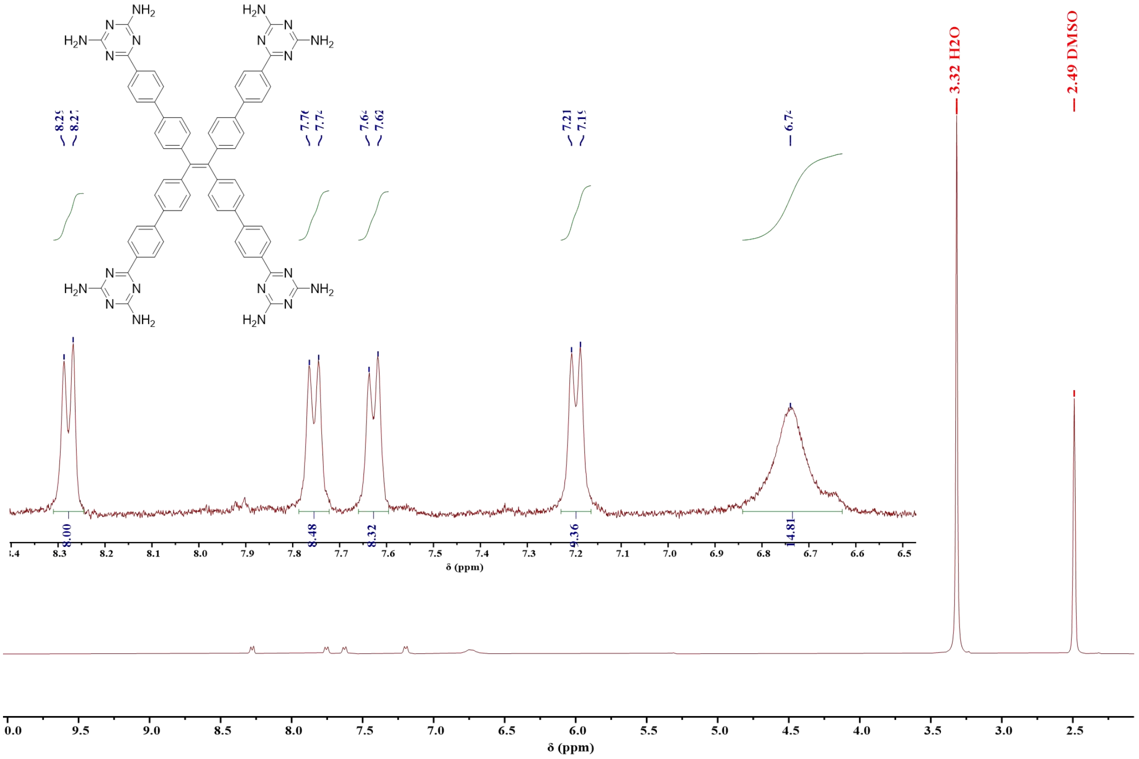

Figure S2. ^{1}H NMR spectra of TPEPhDAT (400 MHz, DMSO-d_6).

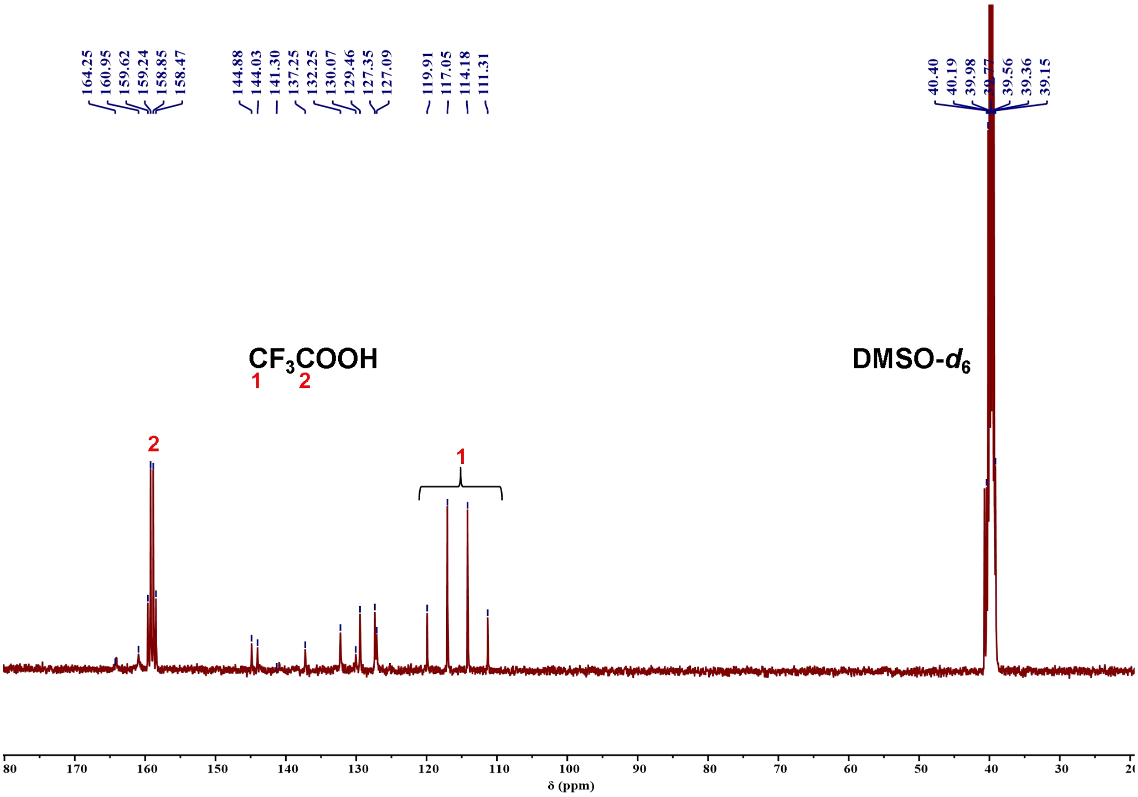

Figure S3. ^{13}C NMR spectra of TPEPhDAT (101 MHz, DMSO-d_6/TFA, v:v = 9:1).

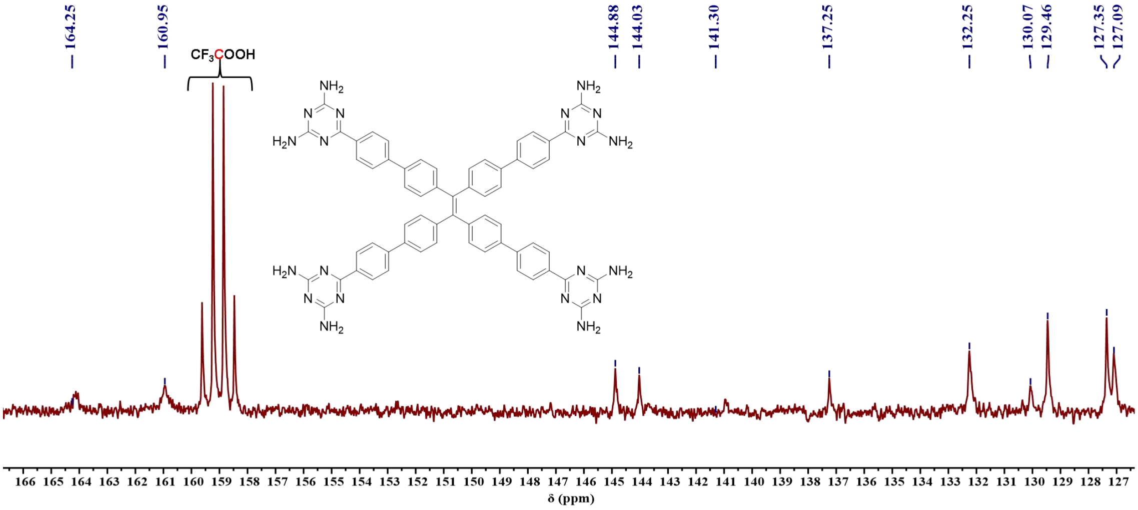

Figure S4. Localized enlarged ^{13}C NMR spectra of TPEPhDAT (101 MHz, DMSO-d_6/TFA, v:v = 9:1).

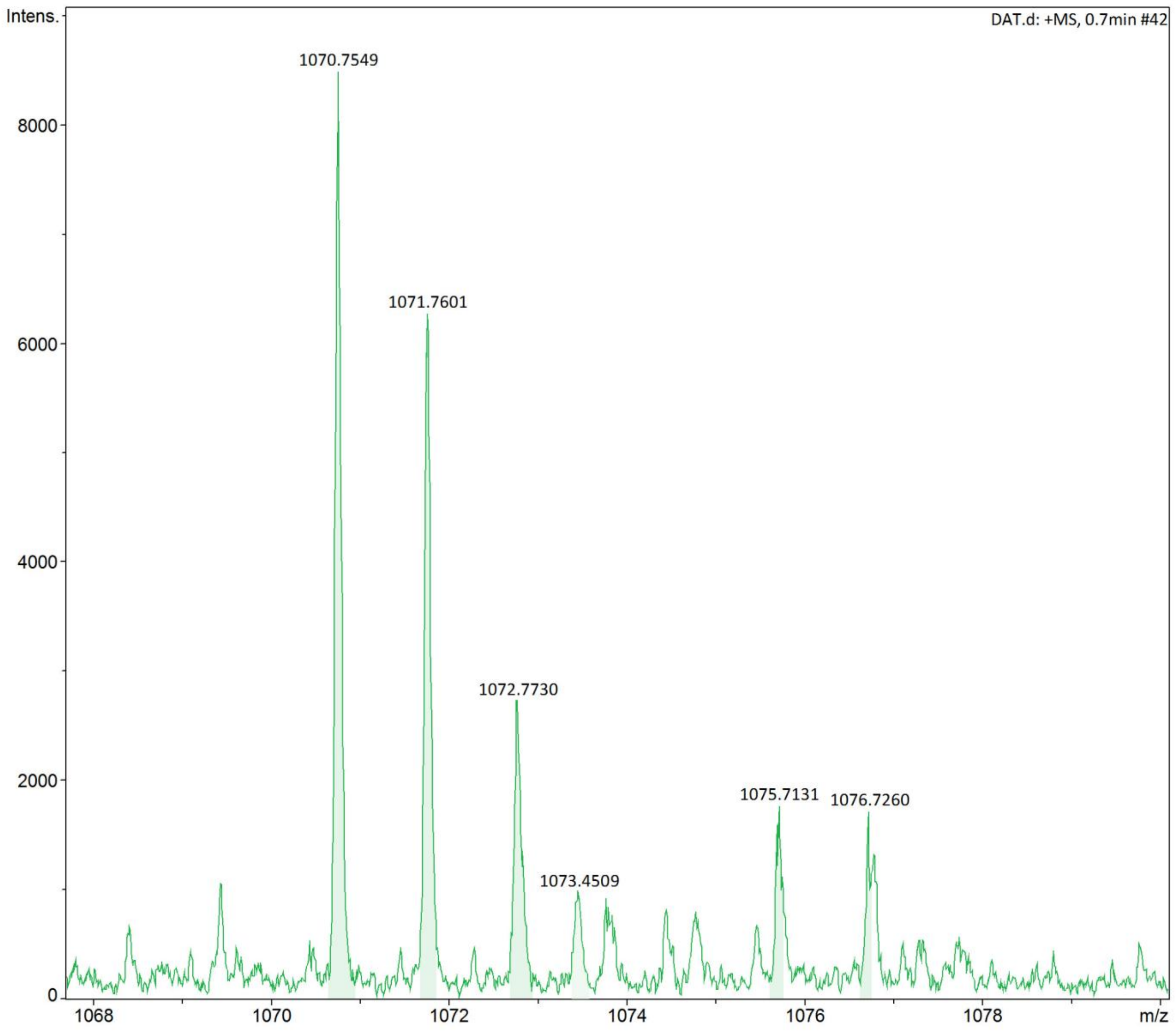

Figure S5. LC-MS spectra of TPEPhDAT.

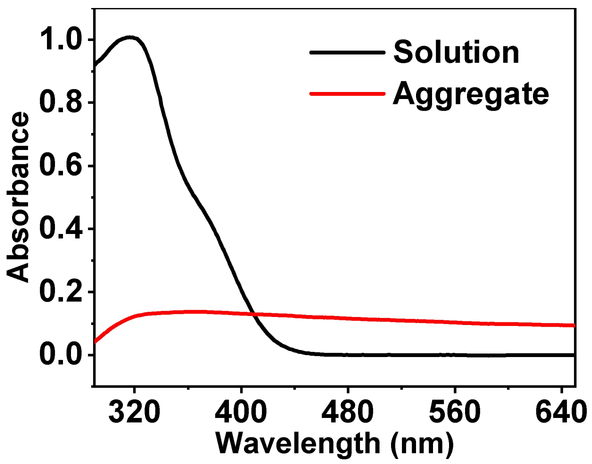

Figure S6. UV-vis absorption spectra of TPEPhDAT in solution (f_{MeOH} = 0 vol%) and aggregate (f_{MeOH} = 95 vol%).

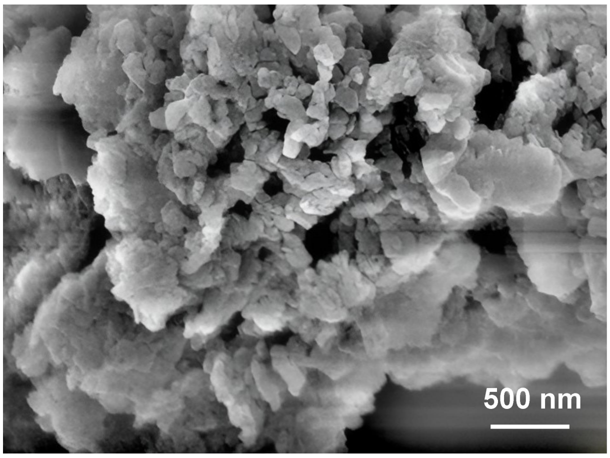

Figure S7. SEM pictures of aggregates at f_{MeOH} = 95 vol%.

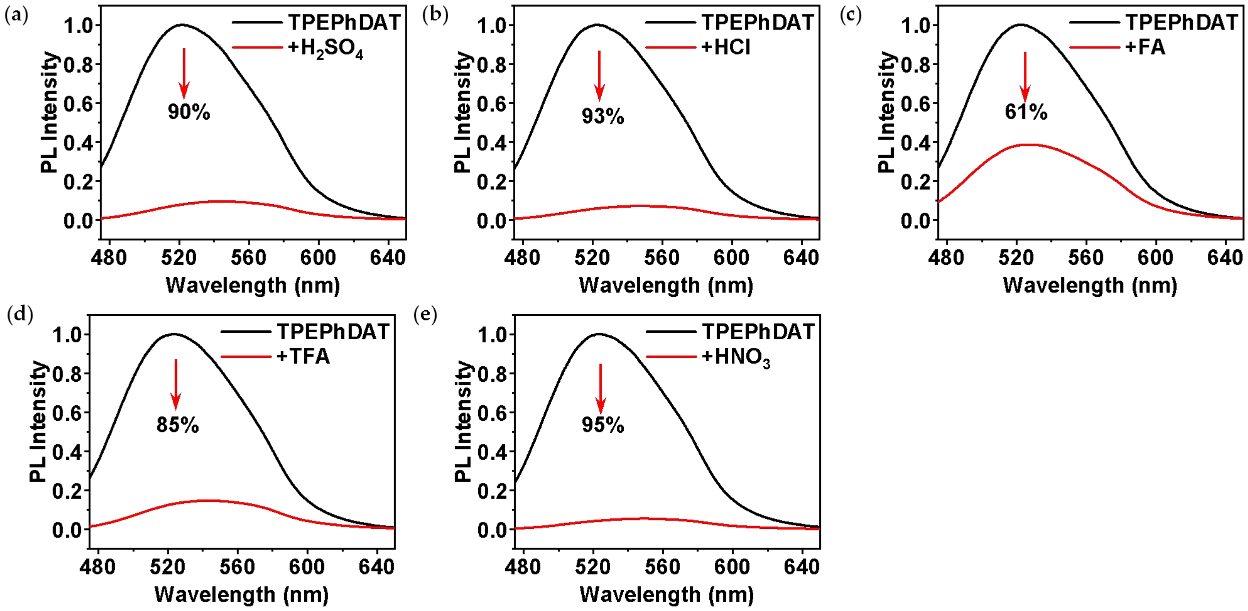

Figure S8. Emission spectra of TPEPhDAT dispersed in water before and after the addition of an aqueous solution of (**a**) H_2SO_4, (**b**) HCl, (**c**) FA, (**d**) TFA, and (**e**) HNO_3.

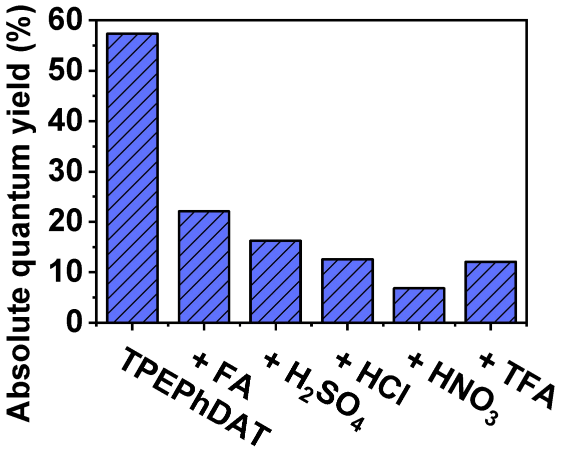

Figure S9. The absolute quantum yield of TPEPhDAT before and after the addition of various acids.

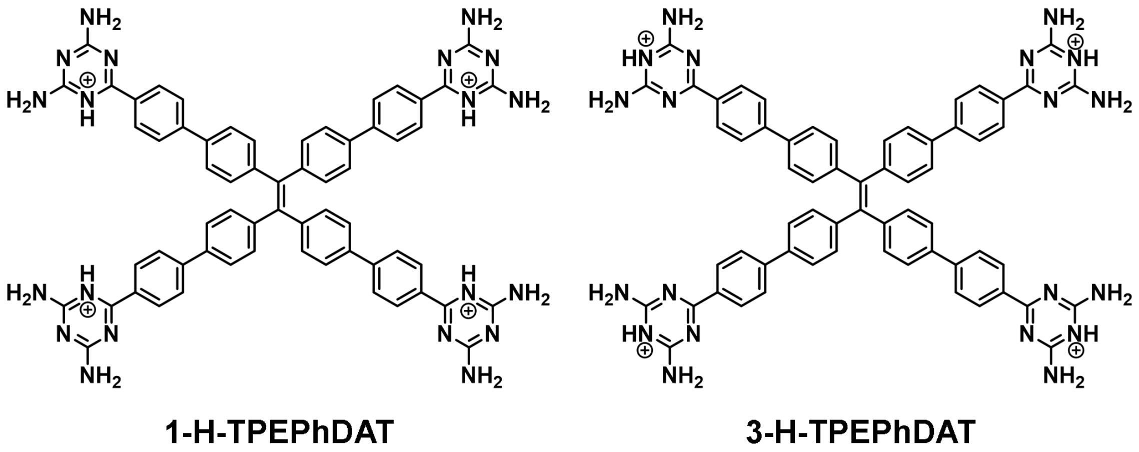

Figure S10. There are two positions where protonation may occur: 1-H-TPEPhDAT and 3-H-TPEPhDAT.

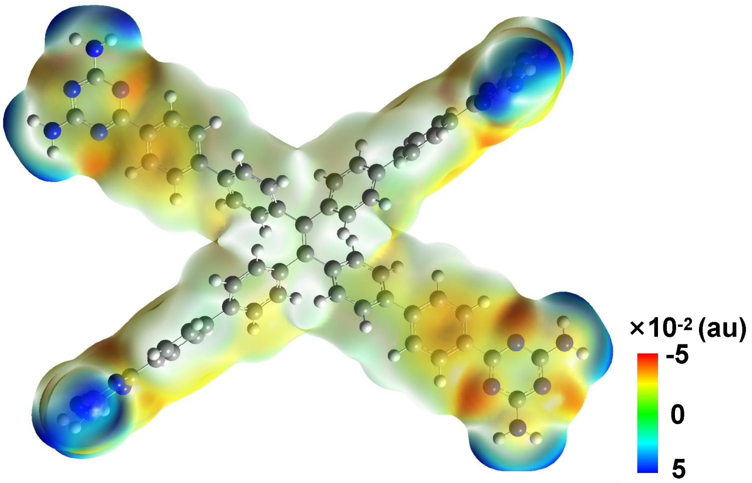

Figure S11. Electrostatic potentials mapped on isodensity surfaces of TPEPhDAT.

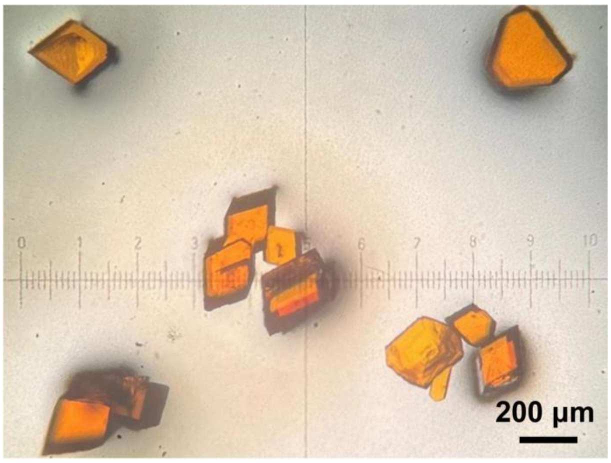

Figure S12. Microscopic optical image for TPEPhDAT-TFA.

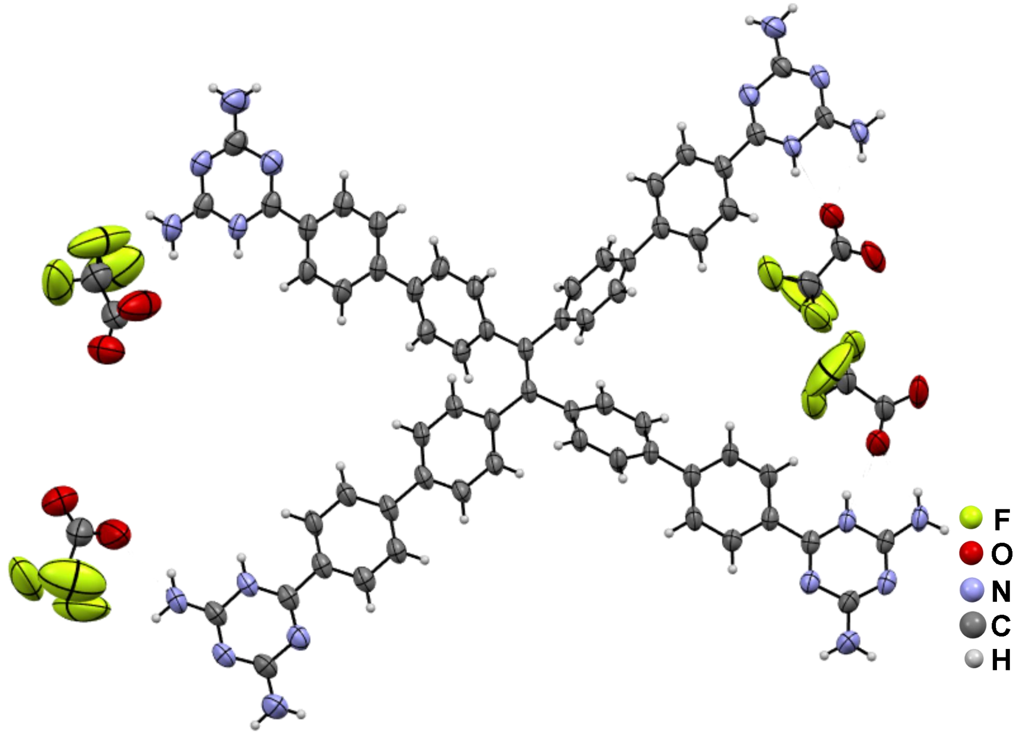

Figure S13. Crystal molecular diagram of TPEPhDAT-TFA (ellipsoids drawn at 50% probability).

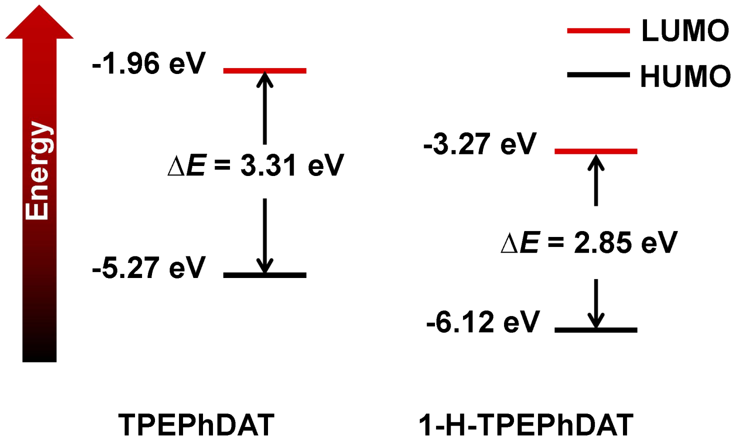

Figure S14. Calculated absorption energy of bared and protonated TPEPhDAT monomer.

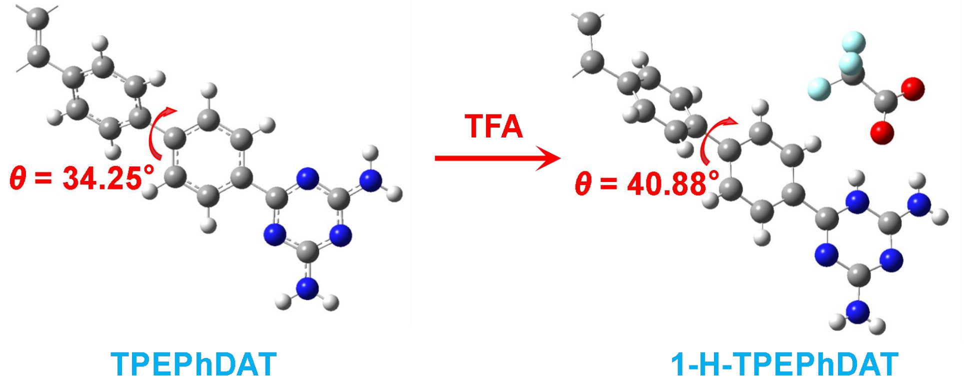

Figure S15. Change in the dihedral angle between two phenyl planes before and after TPEPhDAT protonation.

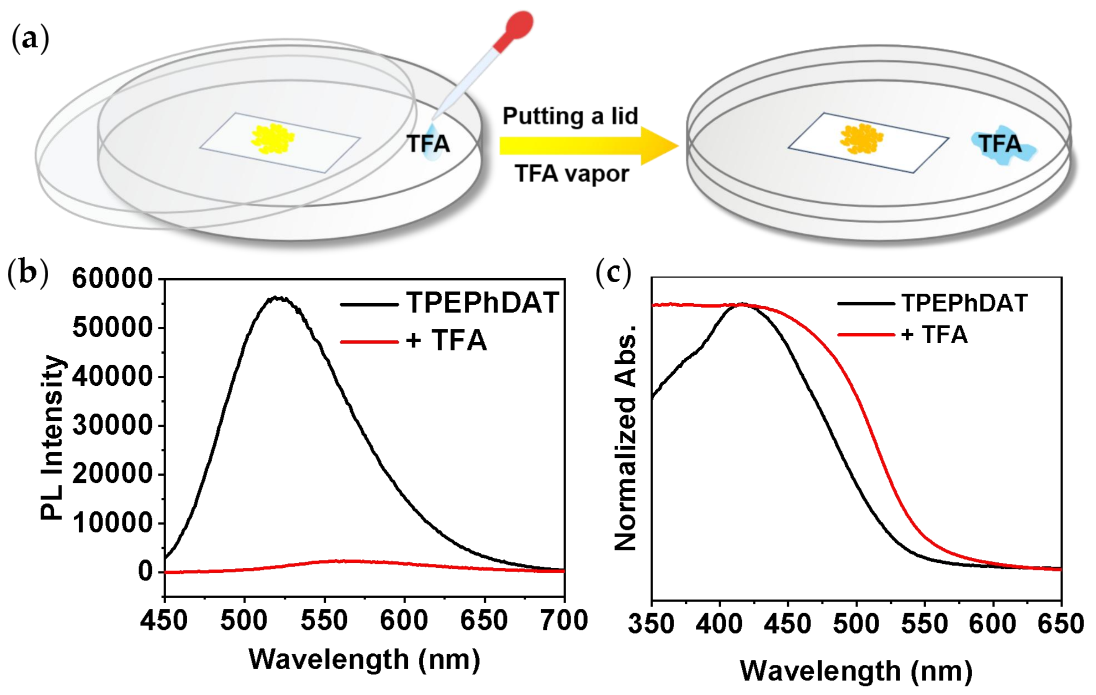

Figure S16. (**a**) Schematic diagram of TPEPhDAT fumigated TFA. Fluorescence spectra (**b**) and UV-vis absorption spectra (**c**) of TPEPhDAT before and after fumigation of TFA.

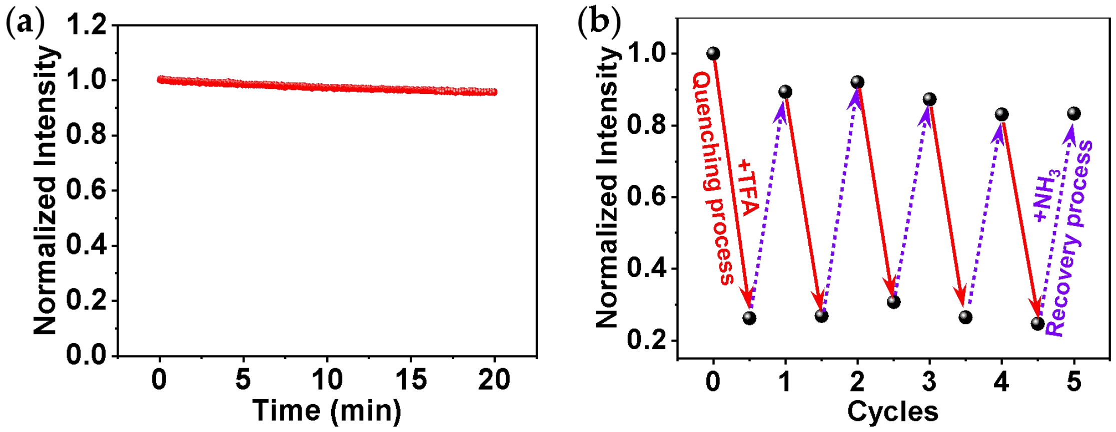

Figure S17. (**a**) The fluorescence emission intensity of TPEPhDAT after continuous UV irradiation for 20 min (λ_{em} = 521 nm); (**b**) Cycle cycles of TPEPhDAT were treated with alternating TFA and NH_3 vapors: the red solid line indicates the quenching process and the purple dashed line indicates the recovery process.
